# Understanding COVID-19 vaccine hesitancy among Black pregnant individuals: a mixed methods study of social media messenger and content type preferences

**DOI:** 10.3389/fpubh.2026.1785021

**Published:** 2026-08-12

**Authors:** Elizabeth Cox, Emma Every, Rachel Johnson, Melissa Baker, Magali Sanchez, Isabelle Crary, Carly Baxter, Simone Stapley, Jeff Munson, Alex Stonehill, Kristina M. Adams Waldorf

**Affiliations:** 1 Department of Health Systems and Population Health, School of Public Health, University of Washington, Seattle, WA, United States; 2 School of Medicine, University of Washington, Seattle, WA, United States; 3 Black Health, Inc., New York, NY, United States; 4 Department of Epidemiology, School of Public Health, University of Washington, Seattle, WA, United States; 5 Department of Communication, University of Washington, Seattle, WA, United States; 6 Department of Psychiatry, University of Washington, Seattle, WA, United States; 7 Department of Obstetrics and Gynecology, University of Washington, Seattle, WA, United States; 8 Department of Global Health, University of Washington, Seattle, WA, United States

**Keywords:** African American, Black, COVID-19, disparity, medical racism, minority, pregnancy, vaccine

## Abstract

**Introduction:**

Pregnant individuals are at increased risk of developing severe illness from coronavirus disease 2019 (COVID-19). They also tend to be vaccine-hesitant, which is even more pronounced in certain racial and ethnic minority groups. The study objective was to determine whether social media advertisements promoting COVID-19 vaccination could influence vaccination likelihood among pregnant and recently pregnant participants who self-identified as Black or African American.

**Methods:**

Participants were interviewed individually or in focus groups to explore their attitudes toward vaccination and were asked to rate their likelihood of receiving a COVID-19 vaccine after seeing a panel of advertisements featuring different messengers (e.g., doctors, peers, older adults, and faith leaders) and content types (e.g., social proof, text-heavy, fear-based, and activation). Advertisement ratings were analyzed using linear mixed-effects models to examine the effects of vaccination status, advertisement messenger, and advertisement content type. Interview data were coded and analyzed to identify qualitative themes. Advertisement ratings differed significantly according to vaccine status, religious affiliation, political affiliation, and marital status.

**Results:**

Participants who were vaccinated, religious, politically conservative, and/or married were more likely to report an increased willingness to receive a COVID-19 vaccine after seeing the social media ads. No specific advertisement messenger or content type was rated as more probable to motivate vaccination. Qualitative analysis identified several themes related to attitudes toward vaccination, including trust in oneself, family, doulas, and faith leaders; mistrust of the government and healthcare system; concerns about medical racism; and personal experiences with individuals recommending or discouraging vaccination.

**Discussion:**

Although no single advertisement messenger or content type was preferred, personal testimony from a trusted source, such as a faith leader or doula, may support vaccine acceptance among Black pregnant women.

## Introduction

The coronavirus disease 2019 (COVID-19) pandemic was associated with vaccine disinformation on social media and other platforms suggesting adverse effects of vaccination on women’s fertility and pregnancy outcomes (1–4). As pregnant individuals were at increased risk of severe illness, morbidity, and mortality from COVID-19, vaccine disinformation threatened their health by increasing the likelihood of vaccine refusal or delay (5–7). Vaccine decision-making during pregnancy is already more complicated than in the non-pregnant state because it involves weighing risks and benefits for both the pregnant individual and the fetus (8). Unsurprisingly, COVID-19 vaccine uptake among pregnant individuals lagged behind that of non-pregnant individuals (6, 9). Public health campaigns leveraging social media can reach millions of people in a cost-effective and rapid manner. Designing effective advertisements that promote vaccination among vaccine-hesitant pregnant individuals requires an understanding of the trusted messengers and content that are embraced by demographic groups (10). Attitudes toward COVID-19 vaccination at the intersection of pregnancy and ethnic minority group membership remain poorly understood, particularly among populations that have historically experienced lower vaccination rates.

Vaccine hesitancy within Black communities has been observed in many studies (9, 11–15). A 2021systematic review and meta-analysis that included more than 100,000 individuals in the United States reported an overall COVID-19 vaccine hesitancy rate of 26.3%, with substantially higher rates among Black (41.6%) and Hispanic (30.2%) individuals (16). COVID-19 vaccine hesitancy is even more pronounced among pregnant individuals, with studies reporting COVID-19 vaccine acceptance rates of only 44–58%, compared to 76.2% in non-pregnant individuals (17–19). Black ethnicity has also been associated with vaccine hesitancy during pregnancy (19). The idea that there is no single universal determinant of vaccine hesitancy within Black communities is supported by qualitative studies that have explored reasons for vaccine hesitancy and mistrust of the vaccine (9, 13, 20, 21). Potential explanations for vaccine hesitancy among Black pregnant individuals include disinformation and inequitable access to vaccination (6, 9). White patients are more likely to have access to a primary care provider and be insured than Black patients (22, 23). Black pregnant individuals are also more likely to experience maternal mortality, independent of insurance status (24–26). Finally, historical acts of medical racism (i.e., the Tuskegee Syphilis Study and the forced sterilization of Black women) represent additional factors that may contribute to general mistrust of healthcare systems (24–26).

The science of public health communication campaigns designed to encourage healthy habits or vaccine uptake is a growing field and has the potential to increase vaccination rates among vaccine-hesitant pregnant populations (10). Several COVID-19 vaccine communication campaigns have included Black communities among their target audiences (27). A social media campaign that reached over millions of users through geo-targeted advertisements featured racially diverse physicians and faith leaders, with one campaign arm directed at ZIP codes with the highest COVID-19 mortality rates and majority Black or Latinx populations (27). This study did not test the likelihood of vaccination across different messengers or content types, but it demonstrated the ability to reach millions of people at a relatively low cost ($17–$26/1,000 people). Another study using semi-structured interviews with pregnant Black women identified positive message framing as a preferred content approach for highlighting the connection between vaccination and infant health (28). Overall, limited data exist regarding which messengers and types of content might be preferred by pregnant Black women in the United States for a vaccine communication campaign.

We hypothesized that pregnant women identifying with a Black racial/ethnic group would report a greater likelihood of vaccination after viewing advertisements featuring specific messengers and content types, as we previously observed among rural White and Spanish-speaking Hispanic pregnant participants in the Western United States (29, 30). We secondarily hypothesized that demographic variables, including vaccination status, political or religious affiliation, and marital status, would be associated with self-reported likelihood of vaccination. We used a mixed methods design to investigate attitudes toward vaccination and how Black pregnant or recently pregnant individuals in urban settings perceive specific messengers and content types in social media ads.

## Materials and methods

### Ethical approval

**Figure 1:**
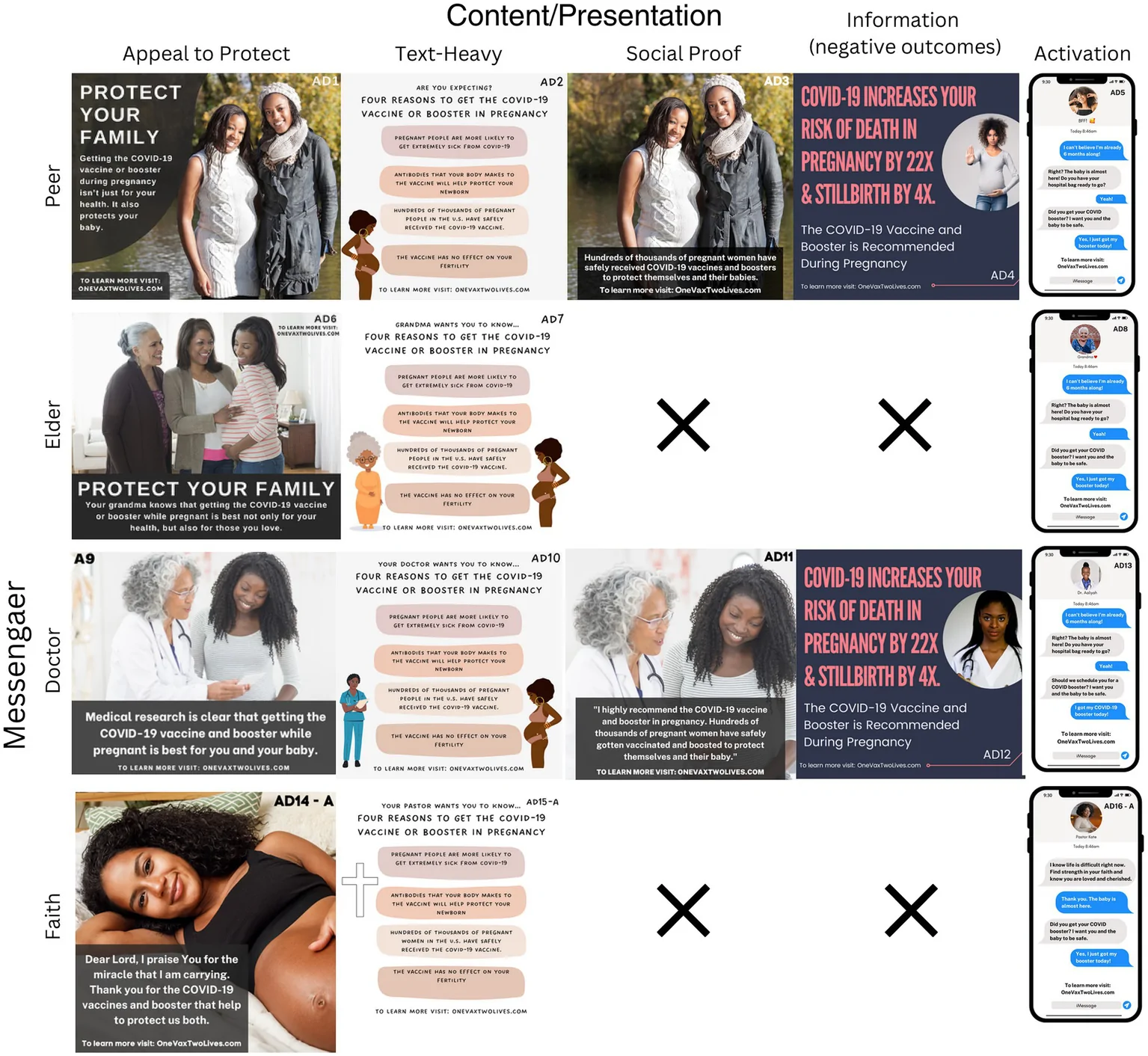
Social media advertisements shown to participants. This figure depicts the social media advertisements shown to participants, organized by messenger and content type. In some cases, combinations of messengers and content were unrealistic (e.g., older adult, social proof), and so these advertisements are indicated by an “X,” as they were not created. Enlarged advertisements are shown in Supplementary Figures S1–S5.

The study was determined to be exempt from human subjects research according to the Common Rule, 45 CFR 46 (Category 2), by the University of Washington Institutional Review Board (STUDY00014356; initial approval 11/05/2021). Informed consent was waived as the study was classified as exempt. A verbal consent process was conducted at the beginning of the survey to describe the voluntary nature of participation, data confidentiality, and participants’ right to withdraw at any time. The images included in Figure 1 were obtained from licensed stock image platforms (Canva Pro, Unsplash, and Pexels), all of which require and provide model releases permitting use in commercial and editorial contexts, including scholarly publication. The individuals depicted are not research participants and were not recruited for this study, and the images were not generated in the context of human subjects research. No identifying personal information is associated with the images, and they are used solely for illustrative purposes.

### Study design

This was a mixed methods study designed to capture both qualitative and quantitative data on vaccine hesitancy and reactions to public health advertisements. We gathered qualitative data via direct interviews and focus groups discussing participants’ experiences with COVID-19 vaccination and pregnancy. The inclusion criteria included being currently or recently pregnant (within the previous 6 months) and self- identifying as Black, Afro-Latinx, or mixed ethnicity with Black or African American ancestry. Quantitative data consisted of individual ratings collected after participants viewed advertisements promoting COVID-19 vaccination.

### Key informant interviews

Key informant interviews shaped the development of the interview facilitator guides for the focus groups. One key informant interview was conducted with a physician specializing in the care of Black pregnant individuals, selected because of their direct clinical experience with COVID-19 vaccine hesitancy in this population and their ability to identify, from a provider perspective, the primary concerns driving vaccine refusal. The key informant interview served a formative rather than evidentiary function. It was conducted at the design stage to inform the development of the focus group guide and the content of the social media advertisements, not to generate primary data for the analysis. In addition, one interview was deemed sufficient given this supplementary function, as the goal was to ground the study instruments in current clinical reality rather than to establish theoretical saturation.

### Patient and public involvement

Feedback from a key informant interview with an obstetrician self-identifying as Black helped shape our research question and focus group questions. We also vetted the focus group questions, focus group guide, and advertisements with several employees of Black Health, Inc. (formerly the National Black Leadership Commission on Health). Feedback from these interviews shaped the questions and study materials. We also worked with Black Health to identify the method of participant recruitment and the format in which study interviews would take place. Focus groups were held during community baby shower events organized by Black Health or its community partners to engage community members in trusted spaces. Dissemination of these research findings will occur through Black Health to its Board and the communities participating in the research.

### Participants and procedures

Our study population included 113 participants from urban areas across the United States who were at least 18 years of age and were currently pregnant or had been pregnant within 6 months of study participation. These urban areas included the Bronx, Brooklyn, Manhattan, Queens, Staten Island, Atlanta, Detroit, Syracuse, and Upstate New York. Black Health recruited participants directly by hosting community “Baby Showers” and partnering with community organizations with a direct link to Black or African American pregnant or recently pregnant individuals, including the Afiya Center, Mothering Justice, and the New York Police Department Community Engagement Office.

### Measures and instruments

Study participants completed a 14-question demographic survey in REDCap that included questions on ethnicity, socioeconomic status, political and religious affiliations, and COVID-19 vaccination status. Participants then completed a 30–45 min direct interview or facilitated focus group discussion focused on their attitudes toward COVID-19 vaccination and risk, trusted sources of information, and health decision-making processes during pregnancy. Finally, participants were shown four sample advertisements promoting the COVID-19 vaccine during pregnancy. They completed an additional REDCap survey that measured their responses to the advertisements. Participants were asked, “After seeing this ad, are you more or less likely to receive the COVID-19 vaccine during your pregnancy or get a booster?” Responses were measured using a self-reported 5-point Likert scale. The scale was anchored at 1 (Strongly agree: I am more likely to get vaccinated) and 5 (Strongly disagree: I am less likely to get vaccinated). Lower scores indicated a greater likelihood of vaccination after viewing the advertisements.

### Advertisement design

To assess respondents’ opinions of social media advertisement messengers and content, we created 19 sample social media advertisements, which tested four types of messengers, such as peer, doctor, older adult, and faith leader, and five types of content, such as activation, social proof, text-heavy, appeal to protect, and information (negative outcomes; Figure 1, enlarged advertisements are shown in Supplementary Figures S1–S5). The selection of messenger types for our advertisements was guided by prior literature on trusted health information sources in Black communities. Peer messengers were defined as individuals who share the demographic characteristics and lived experiences of the target audience. Older adult messengers were included based on qualitative evidence that multigenerational family members, particularly grandmothers and older female relatives, play an important role in health decision-making among Black pregnant women; “older adult” was operationally defined as an older Black woman depicted in the advertisement rather than by participant self-report. Doctor messengers were included because healthcare providers remain among the most commonly cited sources of vaccine information during pregnancy (31). Faith leaders were included because Black churches serve as trusted institutions linking Black communities and public health infrastructure (32–35). All messengers depicted were Black, consistent with the study’s focus on ethnicity-concordant messaging (36). A nuanced approach was used to align faith-based messengers with respondents’ religious affiliations, leveraging data collected earlier in the REDCap survey. This personalized strategy triggered branching logic within the advertisement display, ensuring that religious figures such as priests, pastors, or Imams were presented based on participants’ self-identified religious affiliations.

The content types were selected to represent a range of persuasion strategies, including motivational framing, normative appeals, factual risk communication, and action prompts, that have been studied in vaccine communication research (37). Advertisements that appealed to protection conveyed the message that becoming vaccinated against COVID-19 would help protect the family or the fetus from disease (38). In the text-heavy advertisements, the goal was to provide a short, persuasive list of reasons to get vaccinated. Social proof advertisements conveyed the message that the vaccine had already been safely administered to thousands of other pregnant women (37). Advertisements featuring information on adverse outcomes underscored the increased risk of COVID-19 among unvaccinated pregnant women (39–42). An activation advertisement was designed to encourage or “nudge” participants to receive the vaccine by providing a reminder for someone who had decided to vaccinate but had not followed through (43). Several combinations of content and messenger type were unrealistic and therefore not used (e.g., faith + social proof). Advertisements were designed by aligning imagery and language with the target audience and were vetted with key informants from Black Health. A deliberate effort was made to prominently feature women and healthcare professionals, mirroring the demographics of the intended audience.

Advertisements were randomized into 10 advertisement sets, each showing four advertisements with different combinations of messengers and content types (Figure 1; Supplementary Table S1). To evaluate participants’ reactions to advertisement messengers and content, we asked participants one qualitative and one quantitative question after each advertisement. First, a qualitative question was asked to determine the initial reaction to each advertisement, including what the respondent liked and disliked about it. The quantitative question asked respondents to rate each advertisement on a five-point Likert scale whether they would be more or less likely to receive the COVID-19 vaccine after seeing the advertisement.

### Data analysis

We used a mixed methods approach to analyze qualitative and quantitative data from direct interviews and focus groups (Supplementary Table S2). For the qualitative analysis, each interview was de-identified and blind-coded twice in the software Dedoose using a thematic codebook (Supplementary Table S3) by two independent coders. Following an iterative approach to codebook development, codes were initially developed based on existing knowledge, research, and the interview guide, then revisited and refined to fully and accurately capture emerging themes in the transcripts. Furthermore, two coders independently blind coded all transcripts and met weekly throughout the analysis period to review progress, discuss emerging interpretations, and resolve discrepancies. When disagreements arose over code assignment, which occurred rarely, coders discussed the relevant passage until consensus was reached, drawing on the original interview guide and study aims. For example, statements expressing distrust of the healthcare system were distinguished from statements expressing distrust of government by assessing whether the participant referenced a specific medical interaction or institution (coded as distrust of the healthcare system) versus a broader political or regulatory actor (coded as distrust of government). We did not compute a formal inter-rater reliability statistic, as our analytic approach prioritized iterative consensus building; however, the weekly meeting structure and explicit decision rules ensured a consistent and transparent coding process.

For the quantitative analysis, we examined the effects of messenger type and content type on participants’ likelihood-to-vaccinate ratings using linear mixed-effects models fit with the lme4 and lmerTest packages in R (version 4.5.1). As each participant viewed and rated four advertisements, models included a by-participant random intercept to account for repeated measures; random slopes were not estimated, given the limited number of observations per participant. The Likert ratings were treated as continuous outcomes. Messenger type and content type were modeled as fixed effects in separate models because their combinations were unevenly distributed and some messenger/content pairings were unrepresented; “Peer” and “Activation” served as reference categories, and no covariates were included. Fixed-effect tests used Satterthwaite-approximated degrees of freedom. A second set of models added a message-feature × participant-characteristic interaction to test whether messenger and content effects varied across vaccination status, marital status, religious affiliation, and political affiliation; each interaction was evaluated as an omnibus (multi-d*f*) Type III *F*-test of the full interaction term.

Separately, we tested whether average likelihood ratings differed across participant characteristics. For each characteristic, we averaged each participant’s four ratings and conducted one-way ANOVA, reporting omega squared (*ω*^2^) as a bias-corrected effect size. For characteristics with a significant omnibus effect, pairwise differences among groups were examined using Tukey’s Honest Significant Difference (HSD) test to control the family-wise error rate (*p* < 0.05).

## Results

### Demographics

A total of 113 participants completed direct interviews and focus groups (Table 1). Most participants were Black or African American, married or living with a partner, and held a high school or bachelor’s degree. The majority were pregnant at the time of the study (61.9%) and were vaccinated against COVID-19 (76.3%).

**Table 1:** Demographic characteristics of participants.

Characteristic	Categories	*N* (%)
Ethnicity* (*N* = 92**)	Black or African American	62 (67.4)
	Afro-Latinx	14 (15.2)
	Mixed ethnicity with Black or African American ancestry	7 (7.6)
Pregnancy status (*N* = 97)	Currently pregnant	60 (61.9)
	Pregnant within the previous 6 months	37 (38.1)
Marital status (*N* = 97)	Married	37 (38.1)
	Not married, living with a partner	21 (21.6)
	Not married, not living with a partner	5 (5.2)
	Single	31 (32)
	Other	3 (3.1)
Level of education (*N* = 97)	Some high school	12 (12.4)
	High school education	32 (33)
	Bachelor’s degree	40 (41.2)
	Master’s degree	8 (8.2)
	Trade school	1 (1)
	Prefer not to say	4 (4.1)
Employment status (*N* = 94)	Employed full-time	23 (24.5)
	Employed part-time	30 (31.9)
	Seeking opportunities	22 (23.4)
	Other	12 (12.8)
	Prefer not to say	5 (5.3)
Annual household income (*N* = 96)	<25,000	28 (29.2)
	25,000–50,000	31 (32.3)
	50,000–100,000	20 (20.8)
	100,000–200,000	4 (4.2)
	Prefer not to say	13 (13.5)
Religion (*N* = 107)	Not religious	22 (20.6)
	Christian (protestant)	28 (26.2)
	Christian (catholic)	23 (21.5)
	Christian (any other denomination)	21 (19.6)
	Judaism	1 (0.9)
	Islam	1 (0.9)
	Muslim	3 (2.8)
	Other	3 (2.8)
	Prefer not to say	5 (4.7)
Political affiliation (*N* = 91)	Very liberal	12 (13.2)
	Slightly liberal	19 (20.9)
	Slightly conservative	14 (15.4)
	Very conservative	8 (8.8)
	Prefer not to say	38 (41.8)
Vaccination status (*N* = 97)	Received a COVID-19 vaccine	74 (76.3)
	Not vaccinated	23 (23.7)
Type of COVID-19 vaccine received (*N* = 68)**	Johnson & Johnson	21 (30.9)
	Moderna	17 (23)
	Pfizer	35 (51.5)
	Other	9 (13.2)
Number of boosters received	1	12 (17.6)
	2	5 (7.4)
	3	5 (7.4)
	4	2 (2.9)

*Respondents were able to select more than one ethnicity. **Respondents were able to select more than one type of vaccine received.

### Main effects of advertisement features and subject characteristics

**Figure 2:**
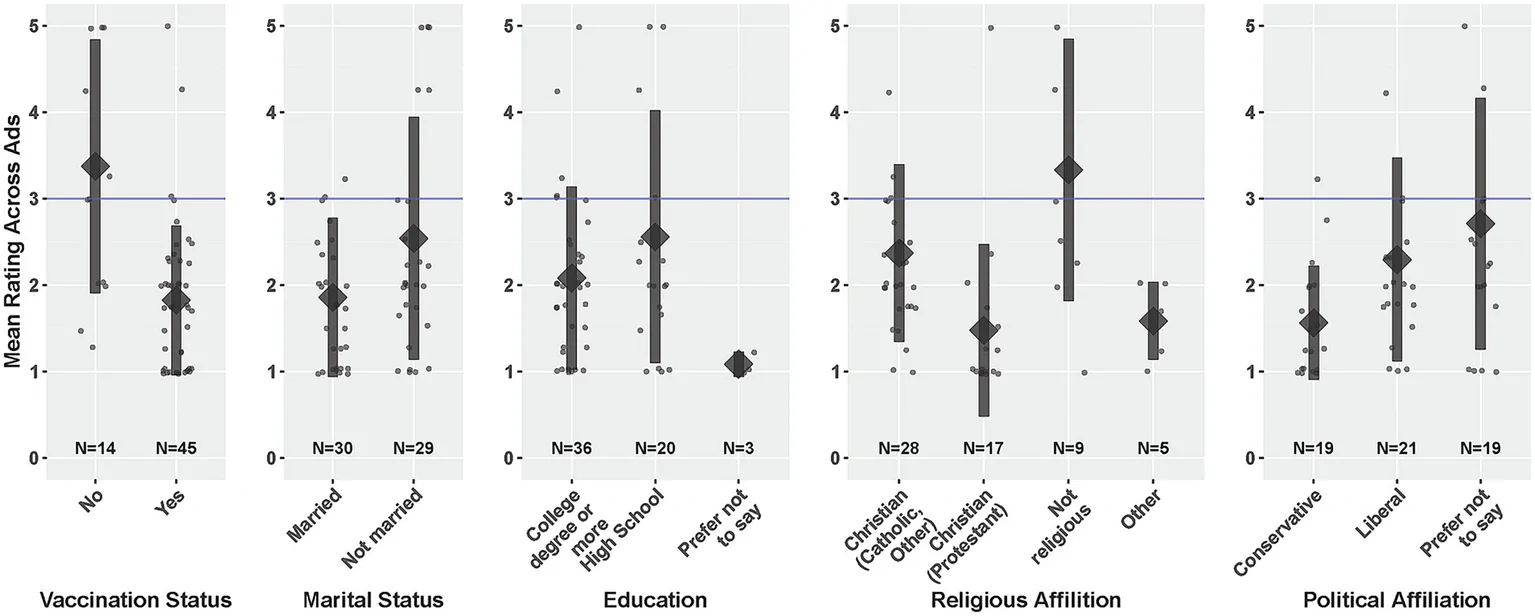
Mean likelihood of becoming vaccinated or boosted by participant characteristic. This figure depicts the mean self-rated likelihood that a participant would receive a COVID-19 vaccine after seeing a collection of advertisements featuring different messengers and content types. The black diamond indicates the overall mean value for each category, and the bar indicates the standard deviation. The Likert scale was constructed so that a rating of one indicated strong agreement that participants were likely to become vaccinated after seeing the advertisement, while a rating of five indicated strong disagreement that they were likely to become vaccinated.

The first set of models found no significant main effects for advertisement messenger or content type, as overall ratings across these dimensions were similar (Figure 2; Table 2, Models 1 and 2). Average ratings by messenger were most favorable for older adult messengers [mean (*M*) = 2.08, SD = 1.36] and least favorable for peer messengers (*M* = 2.24, SD = 1.35). For content, social proof advertisements were most favorable (*M* = 2.03, SD = 1.30), and negative outcome advertisements were least favorable (*M* = 2.32, SD = 1.46). Next, we analyzed the association between participant characteristics/demographic variables and participants’ average likelihood to vaccinate across all messengers and content types. Vaccination status, marital status, religious affiliation, and political affiliation each had a significant main effect on participants’ average likelihood-to-vaccinate ratings (Figure 2; Table 3A). *Post hoc* analyses were conducted to examine specific differences across demographic categories using Tukey’s HSD test to control the family-wise error rate (Table 3B). The analyses revealed that unvaccinated participants and those with no religious affiliation had the highest ratings, disagreeing that the advertisements would make them more likely to vaccinate. In addition, participants who were not married had higher ratings than married participants. Significant pairwise contrasts included Christian (Protestant) versus not religious (*p* < 0.001), Catholic versus Protestant (*p* = 0.044), “not religious” versus “other” (*p* = 0.025), and “conservative” political affiliation versus “prefer not to say” (*p* = 0.009).

**Table 2:** Effect of messenger type and content type on advertisement ratings by vaccine status.

How likely to vaccinate after seeing the advertisement					
Predictors	Estimates	Std. error	Conf. interval	Statistic	*p*-value
Model 1: advertisement messenger type					
(Intercept)	2.25	0.17	1.91–2.60	12.95	<0.001
Messenger [doctor]	−0.04	0.12	−0.27–0.18	−0.39	0.699
Messenger [older adult]	−0.10	0.12	−0.33–0.13	−0.89	0.373
Messenger [faith]	−0.11	0.12	−0.34–0.12	−0.93	0.353
Model 2: advertisement content type					
(Intercept)	2.29	0.17	1.95–2.64	13.12	<0.001
Content [social proof]	−0.09	0.14	−0.35–0.18	−0.63	0.530
Content [text-heavy]	−0.08	0.12	−0.30–0.15	−0.67	0.507
Content [appeal to protect]	−0.18	0.12	−0.41–0.05	−1.53	0.127
Content [fear]	−0.26	0.17	−0.59–0.07	−1.57	0.117

This table illustrates how differences in advertisement messenger and content type had little impact on participants’ ratings of their likelihood of becoming vaccinated after seeing the advertisement. Ratings corresponded as follows: A rating of 1 indicated “strongly agree,” 2 indicated “agree,” 3 indicated “neutral,” 4 indicated “disagree,” and 5 indicated “strongly disagree.” In these models, the intercept was based on the unvaccinated group for the peer messenger (Model 1) and activation content (Model 2).

**Table 3:** Association between participant characteristics and average likelihood of vaccination.

A. Main effects of demographic variables on the likelihood of vaccination (ANOVA)					
Characteristic	Number of groups	*F*	d*f*	*p*-value	Effect size: *η*^2^
Vaccination status	2	24.01	1.57	<0.001***	0.296
Marital status	2	4.94	1.57	0.030*	0.080
Education	3	2.39	2.56	0.101	0.079
Religious affiliation	4	6.70	3.55	<0.001***	0.268
Political affiliation	3	4.89	2.56	0.011*	0.149

B. Pairwise *post hoc* group comparisons (Tukey’s HSD test)				
Group 1	Group 2	Estimate	Conf. interval	*p*-value
*Religious affiliation*				
Christian (catholic, other)	Christian (protestant)	−0.89	−1.77–−0.02	0.044*
Christian (catholic, other)	Not religious	0.96	−0.13–2.05	0.101
Christian (catholic, other)	Other	−0.79	−2.17–0.6	0.441
Christian (protestant)	Not religious	1.86	0.68–3.03	<0.001***
Christian (protestant)	Other	0.11	−1.34–1.56	0.997
Not religious	Other	−1.75	−3.34–−0.16	0.025*
*Political affiliation*				
Conservative	Liberal	0.73	−0.14–1.61	0.118
Conservative	Prefer not to say	1.15	0.25–2.05	0.009**
Liberal	Prefer not to say	0.42	−0.46–1.29	0.49

ANOVA tests compared average likelihood-to-vaccinate ratings, pooled across messengers and content types, across levels of each participant characteristic (Panel A). *F*, *F* statistic; d*f*, numerator and denominator degrees of freedom; effect size is *η*^2^ (eta-squared), representing the proportion of variance in vaccination likelihood explained by the participant characteristic. Tukey’s HSD *post hoc* pairwise comparisons were conducted following each significant omnibus test for participant characteristics with more than two groups (Panel B). Several contrasts reached significance within the religious affiliation category: 1) Christian (Protestant) versus “not religious”, 2) Catholic versus Protestant, and 3) “not religious” versus “other”. The “not religious” group reported the highest ratings, indicating a lower likelihood of vaccination after viewing the advertisements. ***p < 0.001; **p < 0.01; *p < 0.05; ns, not significant.

### Interactions between advertisement features and participant characteristics

Next, we tested whether the within-subject effects of *messenger* and *content* on the rated likelihood were moderated by participant characteristics. For each characteristic, we fit a linear mixed-effects model (a random intercept for participant) containing the message-feature × characteristic interaction and evaluated each interaction using a Type III *F*-test with Satterthwaite-approximated degrees of freedom (R package: lmerTest). As shown in Table 4, none of the messenger × characteristic interactions reached significance, nor did any of the content × characteristic interactions. Thus, the effects of messenger and content on likelihood ratings did not vary systematically across participant subgroups. The strongest signal was a non-significant trend for the messenger × vaccination status interaction, which may warrant follow-up in a larger sample (*p* = 0.07, Table 4).

**Table 4:** Interactions between advertisement features and participant characteristics.

Effect	Sum Sq	Mean Sq	Num d*f*	Den d*f*	*F*-value	*p*-value
Messenger by vaccine status	2.69	0.90	3	163.44	2.4	0.070
Messenger by marital status	0.03	0.01	3	163.41	0.03	0.994
Messenger by religious affiliation	2.89	0.32	9	157.27	0.83	0.589
Messenger by political affiliation	3.15	0.52	6	160.52	1.39	0.221
Content by vaccine status	1.03	0.26	4	163.74	0.67	0.612
Content by marital status	0.36	0.09	4	163.30	0.24	0.918
Content by religious affiliation	6.55	0.55	12	155.47	1.49	0.134
Content by political affiliation	1.32	0.16	8	159.58	0.42	0.905

Each row in the table reports the omnibus Type III *F*-test for a two-way interaction from a separate linear mixed-effects model predicting likelihood ratings, with a random intercept for participant. Messenger and content were within-subject factors, while participant characteristics were between-subject factors. Degrees of freedom were estimated using the Satterthwaite approximation (R package: lmerTest). Sum Sq, sum of squares; Mean Sq, mean square; Num d*f*, numerator degrees of freedom; Den d*f*, denominator degrees of freedom. None of the interactions were statistically significant (all *p* > 0.05).

### Qualitative analysis

The analysis of the interviews and focus groups identified several themes and sub-themes that influenced participants’ decisions about vaccination during pregnancy, including trust/mistrust, personal experiences with someone recommending or discouraging the vaccine, and concerns about medical racism (Supplementary Table S4). Trusting one’s own judgment emerged as a common theme when participants discussed the source they turned to for health information during pregnancy. Respondents described receiving conflicting information and opinions from different sources and reported prioritizing themselves in decision-making. One participant described feeling overwhelmed by the many differing opinions and ultimately relying on her own judgment, stating: “Every doctor in every clinic [has] their own diverse opinions about stuff, get their information from different places, so I just go with what I think is best for me.” Others recounted similarly individualized experiences, stating, “I trust myself,” and “…you just think about what is good for you,” in response to questions about how they make health decisions during pregnancy.

Although some participants described having a trusting relationship with their OB or primary care physician, very few identified a physician as a trusted source of information. In contrast, doulas were trusted sources for some participants, even for those who expressed strong distrust of doctors. When asked whom they trusted for vaccine information, one participant responded, “myself, my doula, my family.” Another participant stated, “If you notice, most doulas are dark skinned, and the government does not like dark skinned people. So that’s who you have to trust.” Other participants expressed trust in their faith leaders. One unvaccinated participant responded to a faith-based activation advertisement by agreeing that, “My pastor is someone I can trust.” Another person stated, “I was sitting in church, and my pastor shared that he been vaccinated. And I say, “Well, God, that’s my sign.” So, I was vaccinated.” Participants trusted a range of sources, with no single messenger consistently preferred. Overall, however, the theme of trust was characterized by reliance on oneself and close relationships, alongside mistrust of, and in some cases fear toward, medical providers. These examples also show how a single personal experience often became a defining moment in a vaccine decision, representing another important theme identified in the qualitative analysis.

Another theme was the influence of medical racism on participants’ lack of trust in the vaccine’s content and manufacturing process. One participant described her community’s concerns about the vaccine: “What I’ve heard from families and friends is that it’s not for Black people. We should not take it because we do not know what’s being put in it. I’ve heard things from about, you know, back in the day, experiments on Black people, right?” This participant specifically noted that a history of experimentation on Black people contributed to her, her family’s, and her friends’ hesitation to trust a vaccine about which they do not have a lot of information. Another participant expressed a similar fear of being experimented on, stating, “I do not trust the government. If we have to pay for a circumcision or ambulance and stuff, why is COVID and flu shots free all year round? They do not care about us.” The fact that the vaccine was offered at no cost made her suspicious of the motivations of those providing the vaccine and of what those institutions might be gaining from vaccination.

## Discussion

This mixed methods study was designed to determine whether a specific advertisement messenger or social media advertisement content type was associated with a greater likelihood of COVID-19 vaccination among Black pregnant and recently pregnant women. No single messenger or type of advertisement content was uniformly preferred. Ethnicity-congruent physician or peer messengers were not preferred, as we observed in prior studies of White and Spanish-speaking Hispanic pregnant (or recently pregnant) women in the Western United States (29, 30). The quantitative data were clearly polarized by vaccination status and religious affiliation, with unvaccinated and non-religious participants rating their likelihood of vaccination as lower after seeing the advertisements, regardless of messenger or content type. The qualitative data indicated themes of trust in oneself, trust in doulas and faith leaders, and lack of trust in the vaccine based on historical medical racism. Obstetrical care is highly sensitive for many patients, and reviews of perinatal and obstetric care among Black women have been largely negative, with reports of racism and bias in healthcare delivery (44–46). Overall, participants’ concerns regarding the vaccine were complex, and trust in the medical establishment was low.

Participants reported many factors contributing to their COVID-19 vaccine hesitancy, including concerns about safety, the speed of vaccine development, and bio-medically inaccurate beliefs. Our advertisements may have failed to persuade vaccine-hesitant individuals because the reasons underlying their hesitancy were diverse and the advertisements did not address their complex concerns. Alternatively, participants were already in a stable, post-decisional state, which was not influenced by any single advertisement messenger or content type. The absence of significant main effects across messengers and content types may, therefore, be understood as a lack of detectable effects under the conditions of this study, rather than as definitive evidence that varying advertisement messengers and content types would have no influence on individuals considering vaccination.

Trusted sources identified through the qualitative analysis included participants themselves, doulas, and, in some cases, faith leaders whose personal testimony was cited as a defining moment in making the decision to accept or refuse vaccination. Whether a social media advertisement featuring a doula would have been more effective than the other messengers tested is unknown but could be a potential direction for future vaccine campaigns. The finding that participants with a religious affiliation had a more favorable attitude toward COVID-19 vaccination suggests a potential role for the Black Church in encouraging vaccination (32, 34, 47). Prior to the COVID-19 pandemic, faith leaders demonstrated the ability to promote influenza vaccination among Black adults (48). Developing genuine partnerships with trusted individuals in the community who can support health promotion is an important public health strategy for pandemic preparedness.

A strength of this study is its focus on the opinions and reactions of Black pregnant individuals to vaccine messaging. This group is underrepresented in research and has the lowest vaccination rates during pregnancy. Another strength is the use of Black community health advocates and partners to conduct focus groups and interviews. These individuals and organizations were chosen as they were trusted within the Black community and were likely to elicit honest responses to the interview questions. However, many of these individuals had backgrounds in community health rather than in research, and follow-up questions to encourage more in-depth responses were not always asked. Furthermore, the interviews were often conducted in batches, and quality could not be controlled in real time. Sometimes, it was difficult to discern exactly how many participants’ voices were captured during the interviews. As a result, there was a mismatch between the number of REDCap surveys (quantitative results for advertisement scores) and the number of participants in the qualitative interviews and focus groups. Recruitment was concentrated in urban settings across the northeastern United States, Atlanta, and Detroit, which strengthens the characterization of vaccine hesitancy among urban Black communities but limits generalizability to rural settings or to Black pregnant individuals with less access to structured community networks, such as the baby shower events used for recruitment. Despite these limitations, we believe that qualitative studies among underserved and vaccine-hesitant populations can provide important insights to refine future public health campaigns.

Polarization of opinions by vaccination status made it difficult to determine whether a single advertisement messenger or content type was particularly influential. Whether recruitment of unvaccinated participants actively considering vaccination would have yielded greater variability in advertisement reactions and more actionable data remains unclear. Investigating the role of faith leaders, and potentially doulas, in vaccine decision-making is important to determine if they might serve as credibility bridges, moral validators, or community accountability figures. Future studies recruiting exclusively unvaccinated participants who are actively considering vaccination may provide a more sensitive assessment of whether specific advertisement designs can influence vaccine intentions.

## Data Availability

The raw data supporting the conclusions of this article will be made available by the authors, without undue reservation.
