# Supplementary material for: Understanding COVID-19 vaccine hesitancy among Black pregnant individuals: a mixed methods study of social media messenger and content type preferences
Source: Front Public Health. 2026 Aug 12;14:1785021. doi: 10.3389/fpubh.2026.1785021 (PMC13506916; doi:10.3389/fpubh.2026.1785021)
Supplement: Supplementary file 1 [file Data_Sheet_1.pdf]

SUPPLEMENTAL INFORMATION

Table S1. Number of Subjects Viewing Specific Combinations of Ad Messenger and Ad Content 2
Table S2. Focus Group Interview Guide 3
Table S3. Thematic Codebook 4
Table S4. Key Themes, Sub-Themes, and Quotes 5
Figure S1. Enlarged Social Media Ads with an Appeal to Protect Message. 6
Figure S2. Enlarged Social Media Ads with a Text Heavy Message 7
Figure S3. Enlarged Social Media Ads with a Social Proof Message 8
Figure S4. Enlarged Social Media Ads with Information About Negative Outcomes 9
Figure S5. Enlarged Social Media Ads with an Activation Message 10

Table S1. Number of Subjects Viewing Specific Combinations of Ad Messenger and Ad Content

Messenger	Ad Number	Content	Number of Views
Peer	1	Appeal to Protect	5
	2	Text-Heavy	5
	3	Social Proof	10
	4	Information (Negative outcomes)	5
	5	Activation	5
Elder	6	Appeal to Protect	13
	7	Text-Heavy	6
	8	Activation	9
Doctor	9	Appeal to Protect	7
	10	Text-Heavy	6
	11	Social Proof	9
	12	Information (Negative outcomes)	6
	13	Activation	2
Faith*	14	Appeal to Protect	4
	15	Text-Heavy	11
	16	Activation	14

*The faith ad was designed to match the participant's self-reported religion: Pastor (Protestant or Any Other Christian Faith), Priest (Catholic), and Imam (Muslim).

Table S2. Focus Group Interview Guide

Question Theme	Questions
Pregnancy	Tell me about how this pregnancy/ postpartum is going.<br>Walk me through your biggest challenges right now.
COVID-19 Vaccine	Tell me what you have heard about the COVID-19 vaccine from family and friends.<br>Please explain why you did or didn't believe [what you have heard].<br>Tell me what you heard about the COVID-19 vaccine from a healthcare worker during your pregnancy.<br>Please explain why you did or didn't believe [what you have heard].<br>For those who are part of a Church or faith-based community, tell me what you have heard about the COVID-19 vaccine from members of your faith-based community? Did you believe it? Why or why not?
COVID-19 Experience	Has anyone known people who have had COVID-19?<br>Describe your experiences with the COVID-19 disease either for yourself or others.<br>How concerned are you (or were you) about catching COVID-19 during your pregnancy?<br>***If concerned***, what were you worried might have happened to you or the fetus? ***If not concerned***, tell me more about why you weren't worried.
Decision Making in Pregnancy	Tell me who you generally trust for health information and why.<br>Walk me through how you made the decision to vaccinate yourself or not vaccinate for COVID-19?<br>Follow-up questions: Was anyone's opinion important in helping you make your decision? Was there anything you read or saw that influenced your decision? Please tell me more.<br>**If unvaccinated, partially vaccinated or unboosted**, do you have plans to get vaccinated or boosted for COVID-19, complete your vaccine series or get boosted?

Table S3. Thematic Codebook

Parent Code	Description
Distrusted Sources of Health Information	Participant mentions distrusted sources of health information
Trusted Sources of Health Information	Participant mentions trusted sources of health information
Experiences with COVID-19 for Self or Others	Participant describes their own experiences having COVID-19, or observing someone (near them) else's experience with COVID
Self-perceived Risk of COVID-19	Participant describes self-perceived risk of COVID-19
Barriers to Vaccination	Participant describes perceived barriers to getting vaccinated
Facilitators/cues to action	Participant describes perceived facilitators to vaccination or cues to action
Vaccine Hesitant	Participant describes hesitancy of vaccines or sources of vaccine hesitancy
Vaccine Positive	Participant describes feeling positively toward vaccines
Interactions with health care provider	Participant describes experiences with health care provider
Experiences with COVID-19 vaccine	Participant describes experiences receiving the COVID vaccine, either for themselves or those around them
Challenges in Pregnancy	Participant describes challenges faced in their pregnancies

Table S4. Key Themes, Sub-Themes, and Quotes

Theme	Associated sub-theme	Supporting Quotes
Trust	Trust in Self<br>Trust in Family<br>Trust in Doulas	"Every doctor in every clinic [has] their own diverse opinions about stuff, get their information from different places, so I just go with what I think is best for me."<br>"I trust myself."
		"My mom used to [tell] me all the time, 'you should get it. You got the opportunity to get it. Go get it."
		In response to who is a trusted source of information: "myself, my doula, my family."<br>"If you notice most doulas are dark skinned, and the government don't like dark skinned people. So that's who you have to trust."
Mistrust	History of Medical Racism<br>Mistrust of Government<br>Mistrust of Healthcare system	"What I've heard from families and friends is that it's not for Black people. We shouldn't take it because we don't know what's being put in it. I've heard things from about, you know, back in the day experiments on Black people, right?"
		"I don't trust the government. If we have to pay for a circumcision or ambulance and stuff, why is COVID and flu shots free all year round? They don't care about us."
Personal experience	Positive Influence<br>Negative Influence	"When my pastor unveiled that he took the vaccine, I took the vaccine."
		"I've actually just went to the doctors, and he was telling us that pediatricians recommend you to get the vaccine. But he said he [the doctor] understands, because he said if his wife was pregnant, he wouldn't push it [the vaccine] or wouldn't want it right now."

Figure S1. Enlarged Social Media Ads with an Appeal to Protect Message.

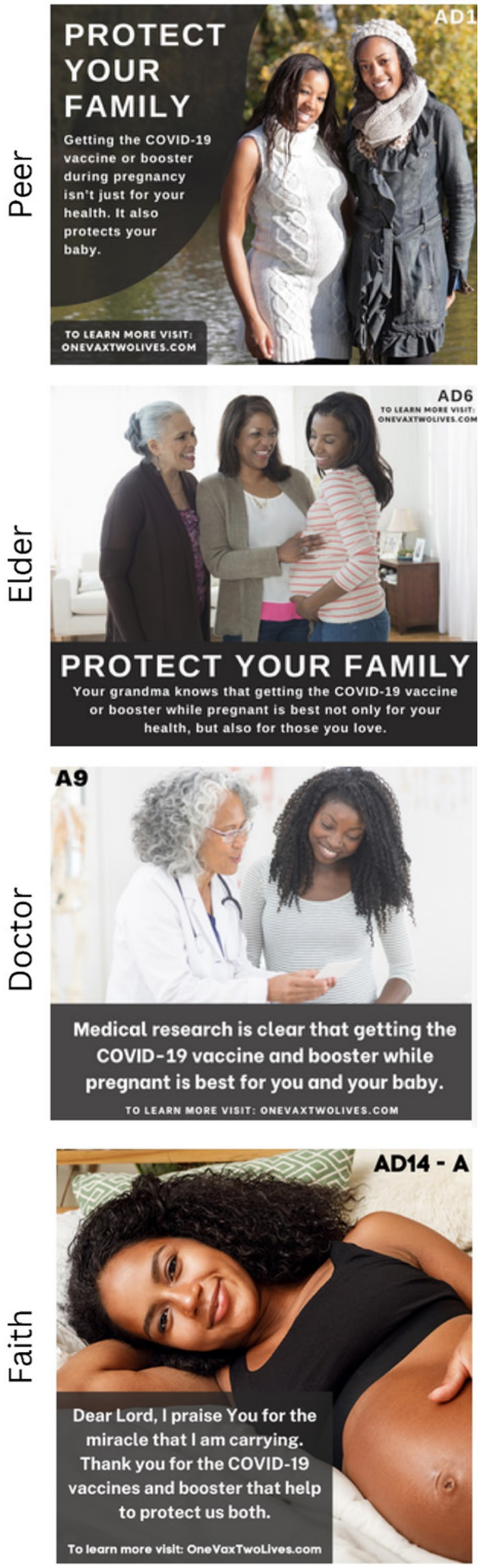

This figure depicts ads featuring different messengers that have appeal to the viewer to protect their family by vaccinating or boosting themselves with the COVID-19 vaccine in pregnancy.

Figure S2. Enlarged Social Media Ads with a Text Heavy Message

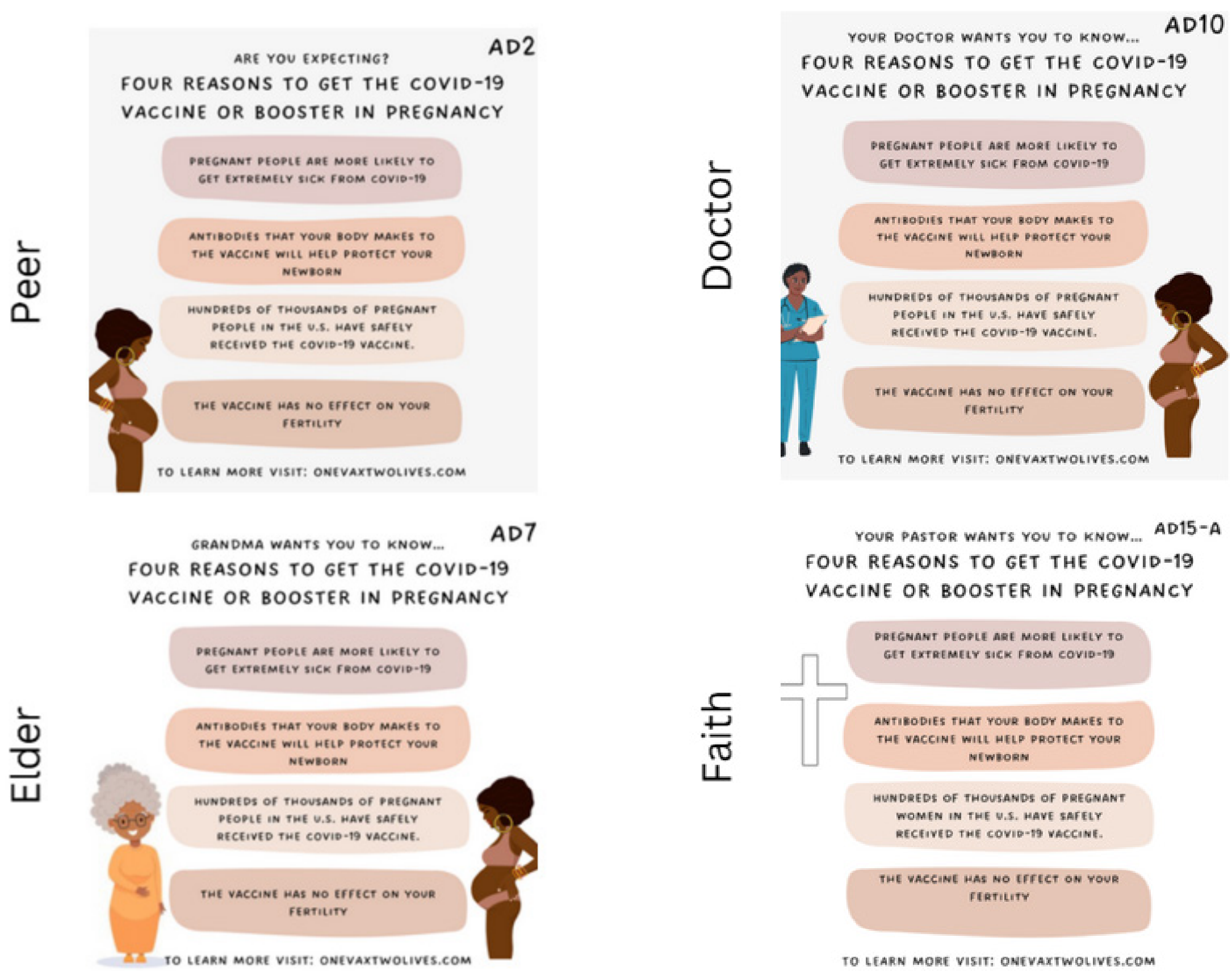

This figure depicts sample social media advertisements featuring a text heavy message promoting the COVID-19 vaccine in pregnancy in combination with different messengers.

Figure S3. Enlarged Social Media Ads with a Social Proof Message

Peer

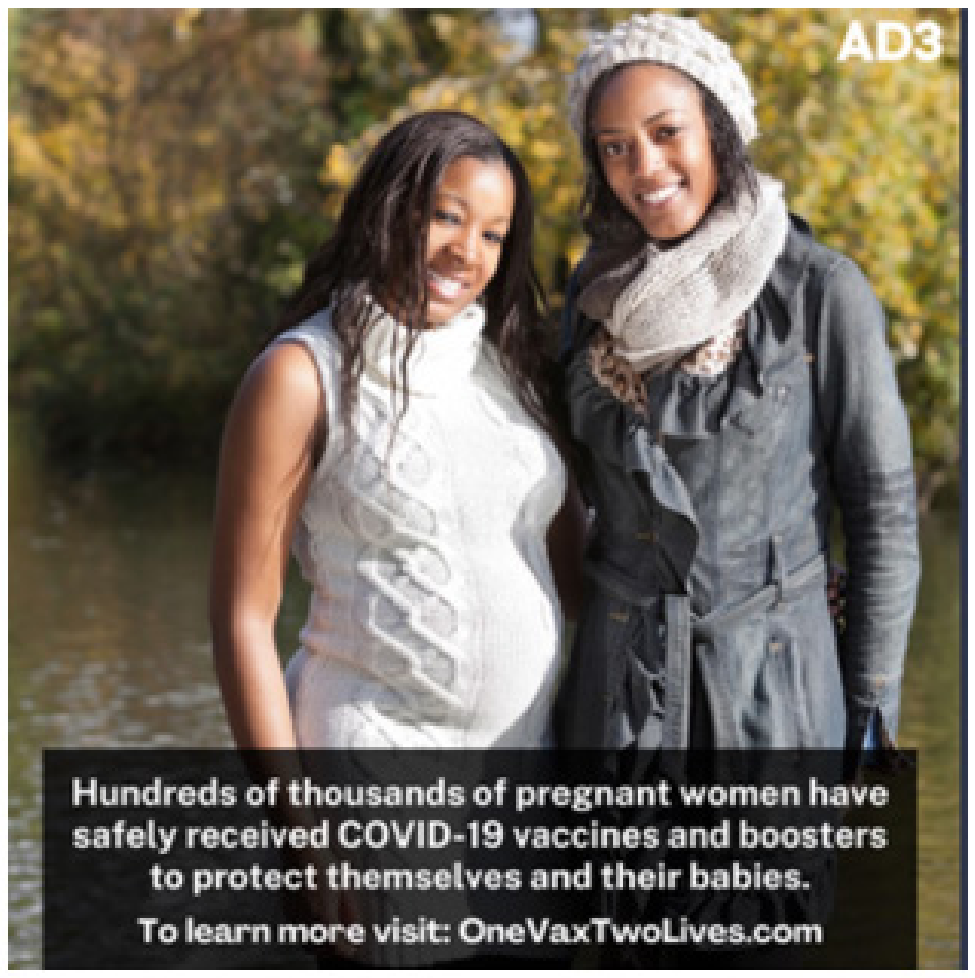

Doctor

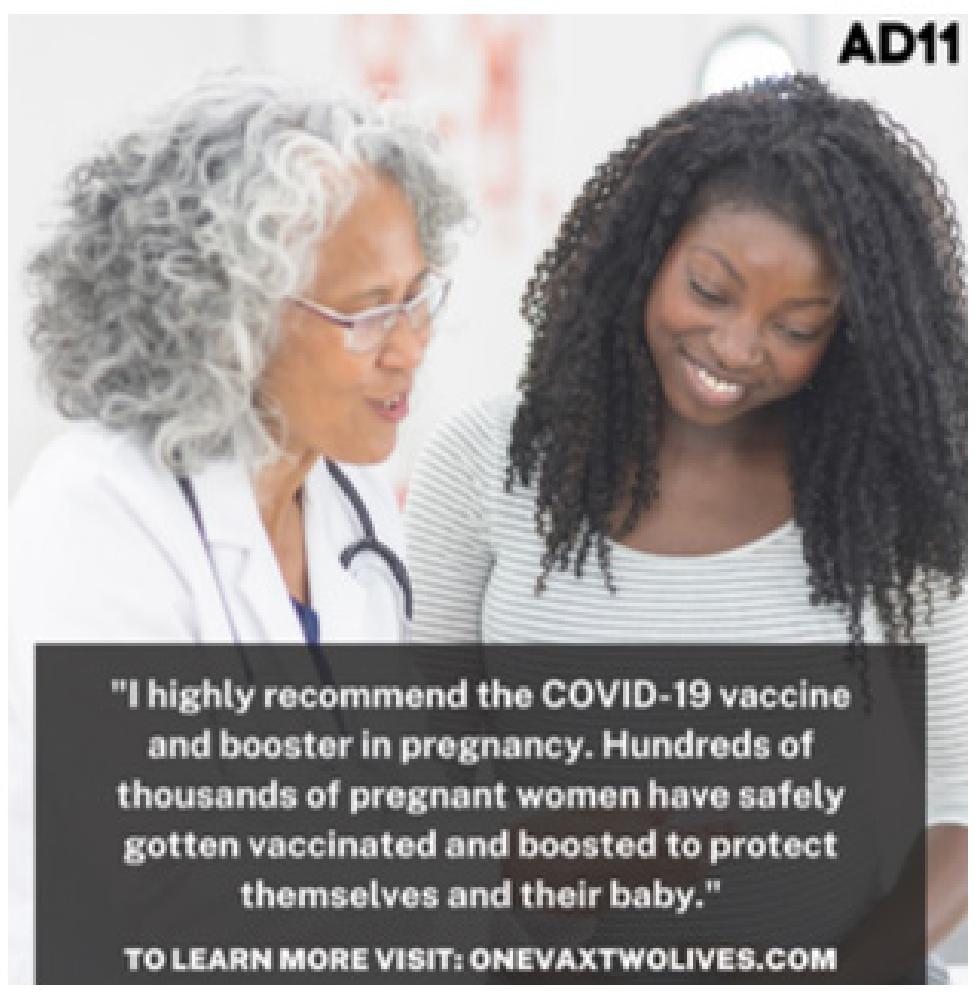

This figure depicts sample social media advertisements featuring a message emphasizing “social proof” by stating that hundreds of thousands of pregnant women had safely received the vaccine. Some messenger combinations were not realistic and were, therefore, not created or shown for this particular ad content type.

Figure S4. Enlarged Social Media Ads with Information About Negative Outcomes

Peer

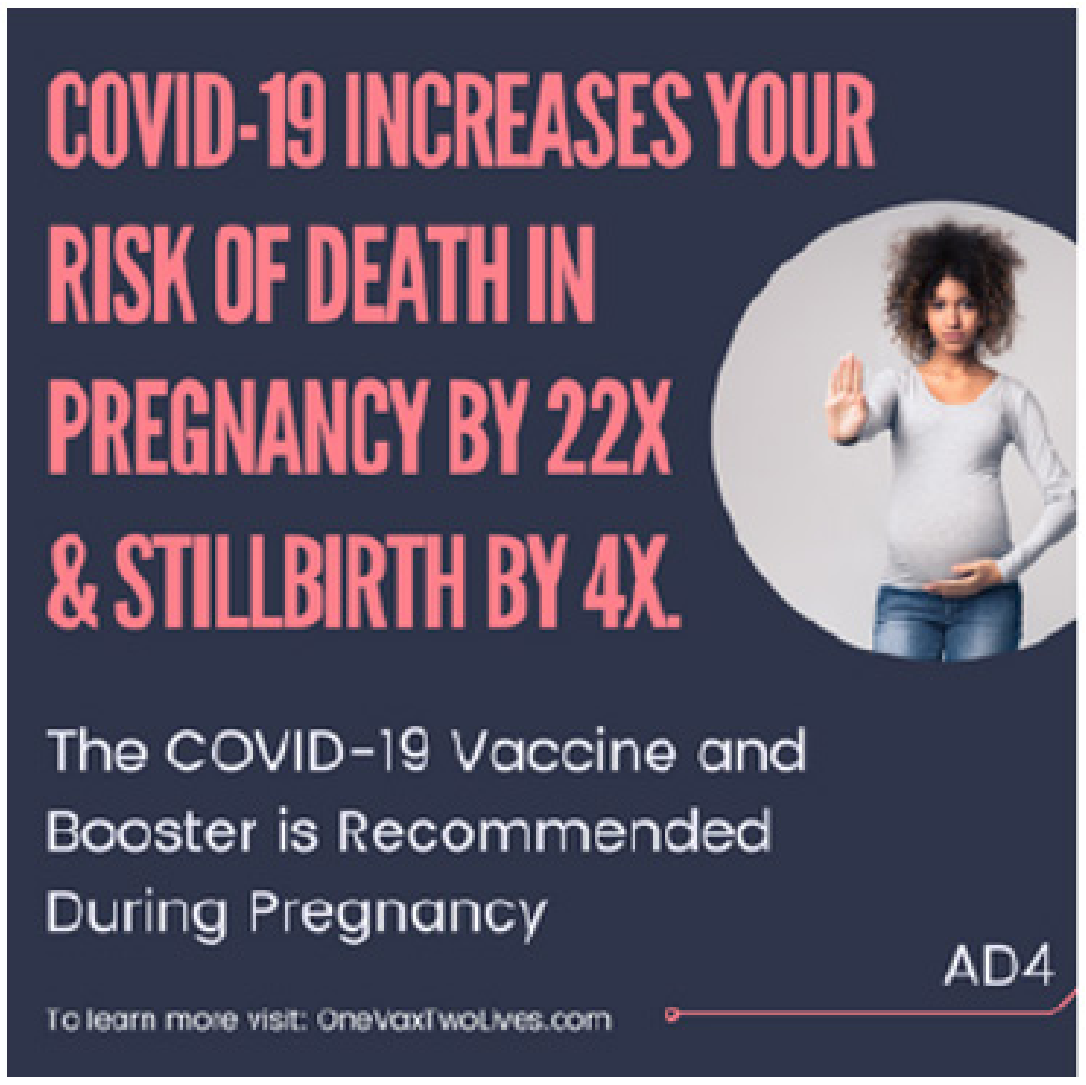

Doctor

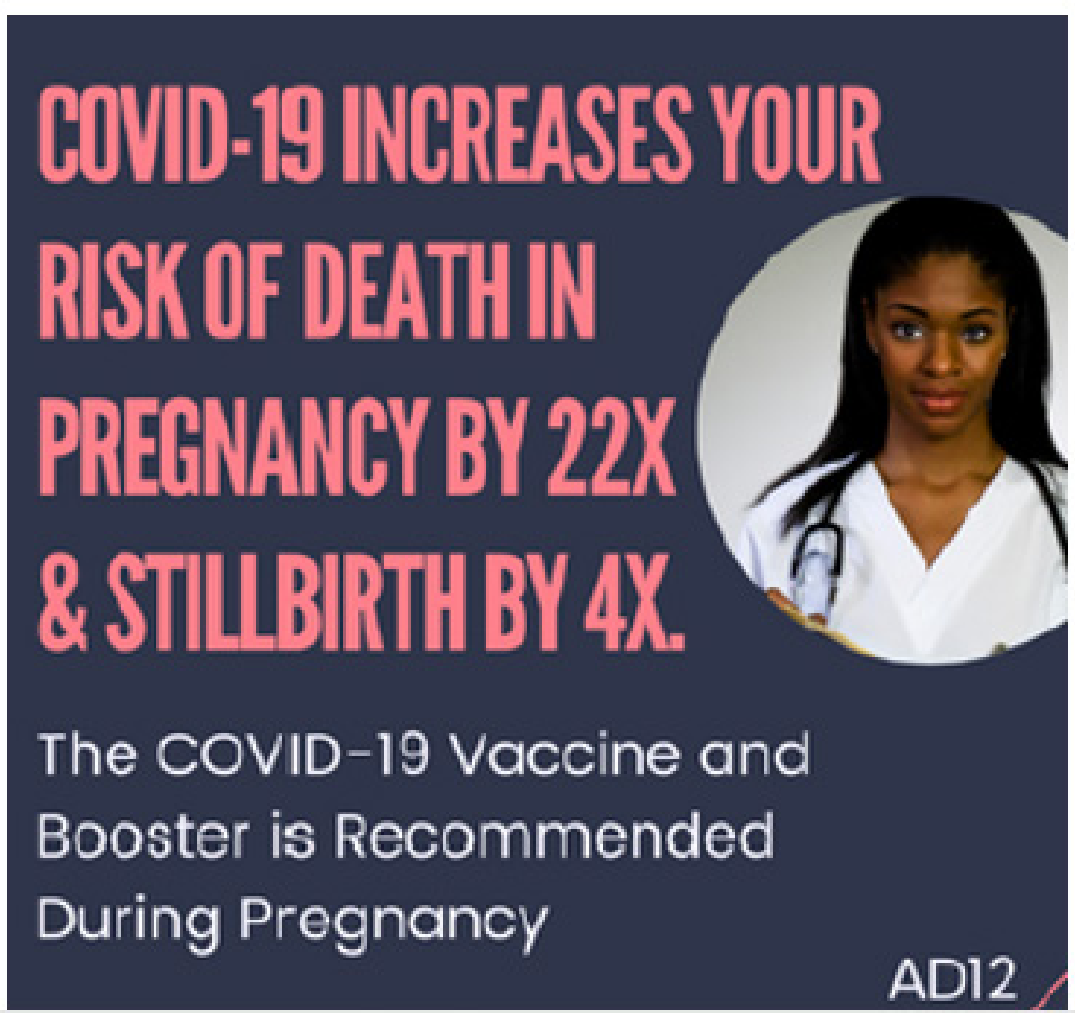

This figure depicts sample social media advertisements featuring messages emphasizing the negative outcomes that can occur if a pregnant person contracts COVID-19. Some messenger combinations were not realistic for this ad and were, therefore, not created.

Figure S5. Enlarged Social Media Ads with an Activation Message

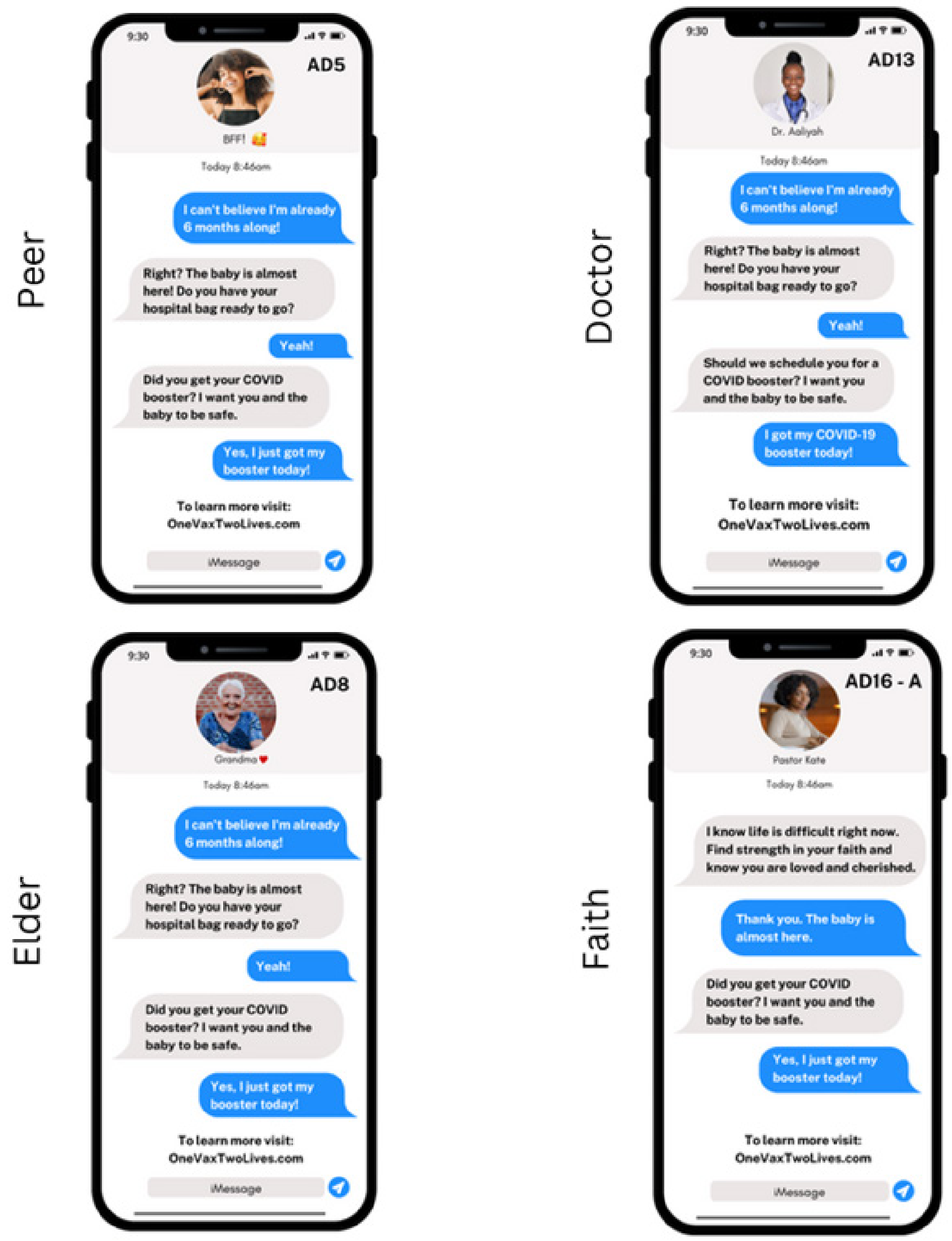

This figure depicts sample social media advertisements featuring an activation message, which are meant to provide an additional prompt to the viewer reminding them that they should get the COVID-19 vaccine.
